# Deep learning predicts the impact of regulatory variants on cell-type-specific enhancers in the brain

**DOI:** 10.1093/bioadv/vbad002

**Published:** 2023-01-12

**Authors:** An Zheng, Zeyang Shen, Christopher K Glass, Melissa Gymrek

**Affiliations:** Department of Computer Science and Engineering, University of California San Diego, La Jolla, CA 92093, USA; Department of Cellular and Molecular Medicine, University of California San Diego, La Jolla, CA 92093, USA; Department of Bioengineering, University of California San Diego, La Jolla, CA 92093, USA; Department of Cellular and Molecular Medicine, University of California San Diego, La Jolla, CA 92093, USA; Department of Medicine, University of California San Diego, La Jolla, CA 92093, USA; Department of Computer Science and Engineering, University of California San Diego, La Jolla, CA 92093, USA; Department of Medicine, University of California San Diego, La Jolla, CA 92093, USA

## Abstract

**Motivation:**

Previous studies have shown that the heritability of multiple brain-related traits and disorders is highly enriched in transcriptional enhancer regions. However, these regions often contain many individual variants, while only a subset of them are likely to causally contribute to a trait. Statistical fine-mapping techniques can identify putative causal variants, but their resolution is often limited, especially in regions with multiple variants in high linkage disequilibrium. In these cases, alternative computational methods to estimate the impact of individual variants can aid in variant prioritization.

**Results:**

Here, we develop a deep learning pipeline to predict cell-type-specific enhancer activity directly from genomic sequences and quantify the impact of individual genetic variants in these regions. We show that the variants highlighted by our deep learning models are targeted by purifying selection in the human population, likely indicating a functional role. We integrate our deep learning predictions with statistical fine-mapping results for 8 brain-related traits, identifying 63 distinct candidate causal variants predicted to contribute to these traits by modulating enhancer activity, representing 6% of all genome-wide association study signals analyzed. Overall, our study provides a valuable computational method that can prioritize individual variants based on their estimated regulatory impact, but also highlights the limitations of existing methods for variant prioritization and fine-mapping.

**Availability and implementation:**

The data underlying this article, nucleotide-level importance scores, and code for running the deep learning pipeline are available at https://github.com/Pandaman-Ryan/AgentBind-brain.

**Contact:**

mgymrek@ucsd.edu

**Supplementary information:**

Supplementary data are available at *Bioinformatics Advances* online.

## 1 Introduction

The World Health Organization estimates that nearly one in six of the world’s population suffers from neurological and psychiatric disorders (World Health Organization, 2006), which comprise 16.8% of global deaths (GBD 2016 Neurology Collaborators, 2019). Large-scale genetic studies have demonstrated that many brain traits and disorders, including Alzheimer’s disease (AD), schizophrenia and intelligence, are highly heritable, and that the majority of genetic variants contributing to these traits reside in non-coding regulatory elements such as transcriptional enhancer regions (Li *et al.*, 2018; Nord and West, 2020). However, identifying the specific genetic variants in these regions contributing to disease is challenging. Compared to protein-coding regions, genetic variants in enhancers are more difficult to interpret. Moreover, enhancer activity is often highly cell-type specific, requiring detailed epigenetic maps to accurately characterize (Li *et al.*, 2018). Further, true causal variants may be in high linkage disequilibrium (LD) with many nearby variants, making it challenging to distinguish between causal versus tagging variants.

Recently, numerous fine-mapping techniques (Benner *et al.*, 2016; Kichaev *et al.*, 2014; Pickrell, 2014) have been developed to prioritize putative causal variants from genome-wide association study (GWAS) data. These methods take as input a list of variants in a trait-associated region of the genome and then quantify the posterior probability of causality of each variant while accounting for local LD structure. However, fine-mapping methods still have difficulty identifying single causal variants with high probability when adjacent variants are highly correlated, or when the density of non-causal variants nearby is high (Schaid *et al.*, 2018). In these cases, prior information such as functional annotations or predictions of the impact of individual variants may aid in variant prioritization (Kichaev *et al.*, 2014).

Multiple recent methods have successfully leveraged deep learning to model regulatory features in non-coding DNA, such as chromatin accessibility and transcription factor binding (Avsec *et al.*, 2021; Corces *et al.*, 2020; Lai *et al.*, 2022; Zheng *et al.*, 2021; Zhou *et al.*, 2019). These methods use model interpretation techniques (Kelley *et al.*, 2016; Selvaraju *et al.*, 2017; Zhou and Troyanskaya, 2015) to generate nucleotide-level annotations of the impact of individual sequence variants on cell-type-specific regulatory features. Here, we develop a pipeline for prioritizing genetic variants predicted to impact cell-type-specific enhancer activities in the brain. We first extend our previously published AgentBind framework (Zheng *et al.*, 2021) to model enhancer activity based on the maps of local acetylation of histone H3 lysine 27 (H3K27ac) measured by chromatin immunoprecipitation sequencing (ChIP-seq) in four brain cell types (Nott *et al.*, 2019) (neurons, microglia, astrocytes, and oligodendrocytes). Our models apply an improved model architecture and incorporate additional spatial information (Liu *et al.*, 2018) in the input data, which we demonstrate boosts model performance. We then apply Grad-CAM (Selvaraju *et al.*, 2017), a post-analytical model interpretation method for neural networks, to compute importance scores at nucleotide resolution and characterize sequence features predictive of H3K27ac activities. We find that variants predicted to have the highest impact on the H3K27ac signals are under stronger negative selection compared to low-impact variants and show a stronger allelic imbalance in observed H3K27ac signals. Finally, we integrate our scores with fine-mapping results from GWAS of eight brain-related traits to demonstrate how our pipeline can identify and characterize putative causal variants that may act via modulating enhancer activity.

## 2 Results

### 2.1 Modeling H3K27ac-enriched enhancers in brain cell types

**Fig. 1.**
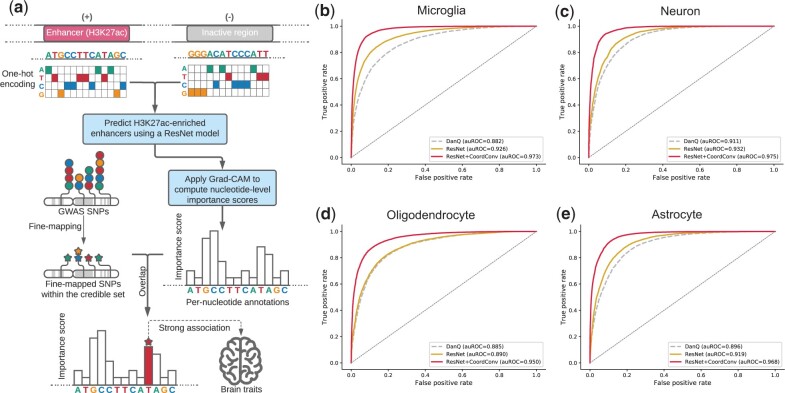
Study overview. (**a**) Method schematic. We construct a ResNet model for four brain cell types and learn sequence features unique to the transcriptional enhancers in these cells. We use Grad-CAM to score the contribution of each nucleotide to model predictions of enhancer activities. In parallel, we fine-map the GWAS SNPs of multiple brain traits and identify the SNPs most strongly predicted to be causal. These candidate causal SNPs are then overlapped with the importance scores predicted by Grad-CAM in relevant cell types for each trait. (**b**–**e**) CoordConv and ResNet improve model performance in (b) microglia, (c) neurons, (d) oligodendrocytes and (e) astrocytes. In each plot, receiver operator curves (ROC) are shown for H3K27ac predictions generated from DanQ (grey dashed line), ResNet (golden solid line) and ResNet + CoordConv (red solid line)

We obtained published H3K27ac ChIP-seq data and ATAC-seq data for four brain cell types (microglia, neurons, oligodendrocytes and astrocytes) (Nott *et al.*, 2019) (Fig. 1a; Section 4). For each cell type, we identified putative active enhancers as H3K27ac-enriched open chromatin at intronic and intergenic regions. After filtering, our dataset consisted of between 12 074 and 21 415 non-overlapping putative active enhancers for each cell type (Supplementary Table S1). For each cell type, we constructed a binary dataset consisting of 1 kb sequences centered at H3K27ac-enriched enhancers (positive samples) and sequences sampled with matched GC-content distributions (negative samples). We created multiple copies of each sample through window shifting (Section 4) in order to reduce model overfitting and to ensure model predictions are robust to the relative location of H3K27ac signals within each sequence.

We trained a separate model for each cell type. Our model training process consisted of two steps: pre-training and fine-tuning. Previous studies (Novakovsky *et al.*, 2021; Zheng *et al.*, 2021) have found that pre-training could substantially improve the performance of deep learning models in modeling genomic sequences, especially for small datasets. Similar to AgentBind, we first pre-trained our models using a large published dataset consisting of epigenomics profiles across 35 different cell types available from the DeepSEA project (Zhou and Troyanskaya, 2015). Next, for each brain cell type, we fine-tuned the pre-trained model to predict the active enhancers. Model performance was evaluated using the area under the receiver operating characteristic curve (auROC) and the area under the precision–recall curve (auPRC). Similar to previous work (Zhou and Troyanskaya, 2015), we left out sequences on chromosome 8 for cross-validation and sequences on chromosome 9 for testing.

We tested two different deep learning architectures: the DanQ (Quang and Xie, 2016) architecture used in AgentBind, and a version of ResNet (He *et al.*, 2016) modified from ChromDragoNN (Nair *et al.*, 2019). This ResNet architecture consisted of five convolutional layers followed by eight residual blocks and two fully connected layers (Section 4). The output of both models was a single number between 0 and 1, indicating how likely the input sequence is to have a strong level of H3K27ac. Compared to DanQ, the ResNet architecture resulted in an average auROC increase of 0.023 and auPRC increase of 0.025 (Fig. 1b–e; Supplementary Table S2).

Similar to previous studies (Novakovsky *et al.*, 2021; Zheng *et al.*, 2021), we found that the pre-training step was able to boost the performance of our models by 0.041 (auROC) and 0.041 (auPRC) on average (Supplementary Table S2). On top of the ResNet model, we also applied the CoordConv (Liu *et al.*, 2018) technique which adds an extra coordinate channel to the input of the first convolutional layer to better encode spatial information in the input sequences (Section 4). We found this resulted in a notable performance boost, with an average auROC and auPRC increase of 0.050 and 0.051, respectively. Overall, our final models could predict H3K27ac-enriched enhancer regions with high accuracy (mean auROC = 0.966, mean auPRC = 0.967; Fig. 1b–e; Supplementary Table S2).

### 2.2 Identifying sequence features predictive of H3K27ac-enriched enhancers

**Fig. 2.**
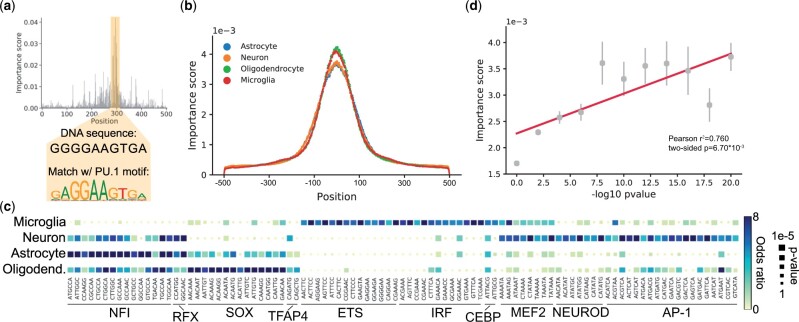
Interpreting sequence features contributing to brain cell-type-specific enhancer activity. (**a**) Example importance score profile. The *x*-axis denotes the central 500 bp region of a 1 kb sequence (chr2:127 885 699–127 886 699). The *y*-axis shows the importance score of each nucleotide predicted from the ResNet+CoordConv model in microglia. In this example, a short DNA sequence that matches the known PU.1 motif is annotated with high importance scores. The components (6-mers) of this motif are enriched in top-scoring 6-mers for microglia shown in Figure 2c. Full score profiles can be viewed through this online IGV link: https://tinyurl.com/ya2rc6nu. (**b**) Aggregate importance score profiles. For each cell type, we computed the average absolute value of the importance score (*y*-axis) per position (*x*-axis) in active enhancer sequences from the ResNet+CoordConv models. (**c**) Top 6-mers enriched for high importance scores across the four brain cell types. The heatmap shows the enrichment of each 6-mer in regions with the highest importance scores for each cell type. The color of each block indicates the odds ratio of the enrichment and the size indicates the two-sided *P*-value based on a Fisher’s exact test. In this heatmap, we only display 6-mers with odds ratio >4 in at least one cell type. (**d**) The relationship between allelic imbalance *P*-values and importance scores. Points show the average importance scores (*y*-axis) for variants in different bins of -log10 *P*-values for allelic imbalance based on ATAC-seq in microglia. Error bars show ±1 standard error. The best-fit line for the points (in gray) is shown as the solid line (in red)

We next applied Grad-CAM (Selvaraju *et al.*, 2017), a model interpretation technique we and others previously used to interpret genomic sequence features learned by deep learning models (Chen *et al.*, 2021; Zheng *et al.*, 2021), to characterize key sequence features contributing to predictions of enhancer activities. We used Grad-CAM to assign nucleotide-level scores quantifying the importance of each base pair to the model prediction (Section 4). An example score profile for a single sequence is shown in Figure 2a. In this example, Grad-CAM scores highlight a short DNA sequence corresponding to a PU.1 motif as an important predictor of enhancer activity of this locus in microglia. Across all four cell types, the majority of high importance scores are concentrated in a 200 bp window around the center of H3K27ac-enriched enhancers (Fig. 2b).

Next, we used importance score profiles to identify sequence features most predictive of H3K27ac-enriched enhancers for each cell type. To this end, we applied two strategies. First, we extracted 6-mers from positive sequences and tested whether each unique 6-mer is enriched within the 6-mers with the highest importance scores (Section 4; Fig. 2c). We used Tomtom (Gupta *et al.*, 2007) to associate 6-mers with known motifs. Tomtom compares each 6-mer against the motifs in the JASPAR database (Castro-Mondragon *et al.*, 2022), a database with a large collection of common transcription factor binding motifs, and then ranks motifs based on their alignment with the query sequence. The most predictive 6-mers are highly cell-type specific. For example, the top 6-mers for microglia are associated with ETS, IRF, CEBP and MEF2 motifs, which correspond to well-documented transcription factors important for microglia phenotype and function (Gosselin *et al.*, 2017; Holtman *et al.*, 2017; Masuda *et al.*, 2012). Further, 6-mers corresponding to the motifs for NeuroD transcription factors are most strongly enriched in neurons, consistent with the known role of these factors in neuronal differentiation (Tutukova *et al.*, 2021). On the other hand, several motifs are shared across cell types, as exemplified by 6-mers matching the NFI motif in astrocytes, neurons, and oligodendrocytes. Different members of the NFI family have been reported to play an important role in the development of these cell types in both mice and humans (Chen *et al.*, 2017; Wilczynska *et al.*, 2009).

Second, we applied TF-MoDISco (Shrikumar *et al.*, 2018), which leverages per-base importance scores to infer enriched motifs. Compared to our k-mer-based approach above, TF-MoDISco does not require motifs to be a fixed length and also does not require exact matches between sequences from the same motif. The top motifs (motifs occurring in >1% of enhancers) identified from TF-MoDISco are consistent with those inferred from enriched 6-mers (Supplementary Fig. S1) while TF-MoDISco further reveals more long motifs, such as CTCF enriched in astrocytes and oligodendrocytes, which could not easily be captured by our k-mer approach. To test whether identified sequence features are simply related to open chromatin, rather than specifically to enhancer activity, we repeated model training and motif analysis while constraining both positive and negative sequences to be within open chromatin regions (Supplementary Table S2). We found that the enriched motifs discovered by TF-MoDISco were largely similar, although multiple new sequences arise as top enriched motifs, such as RUNX in microglia and oligodendrocytes (Supplementary Fig. S2).

To evaluate our nucleotide-level importance scores, we examined them against allelic imbalance based on microglia ATAC-seq data from 16 individuals (Section 4). Briefly, an imbalance of ATAC-seq reads from each allele at a heterozygous single-nucleotide polymorphism (SNP) indicates a bias in regulatory activity between the two genome copies. We found that nucleotide-level importance scores computed based on the microglia model are correlated with allelic imbalance summary statistics (-log10 two-sided *P*-values based on a binomial test; Pearson *r*^2^ = 0.760; two-sided *P* = $6.70*10^{-3}$; Fig. 2d) and with allelic imbalance ratios (Pearson *r*${}^{2}$ = 0.876; two-sided *P* = $1.97*10^{-3}$; Supplementary Fig. S3), whereas *P*-values computed for other cell types do not show significant correlation with the importance scores from the microglia model (Supplementary Fig. S4; two-sided *P*-value for neurons, oligodendrocytes and astrocytes are respectively 0.80, 0.50 and 0.51). Further, SNPs with low allelic imbalance *P*-values (two-sided *P*$<10^{-10}$) are strongly enriched with high importance scores (top 5% of Grad-CAM scores; two-sided Fisher’s exact test *P* = $2.48*10^{-22}$, odds ratio = 2.80). These results indicate that our importance scores are indeed identifying individual variants with an impact on cell-type-specific enhancer activity.

### 2.3 Variants with high impacts on enhancer activity are under purifying selection

**Fig. 3.**
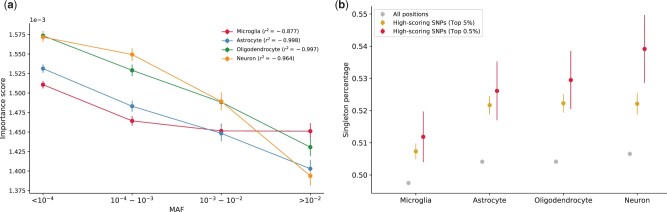
Variants predicted to have high impacts on brain enhancer activity are under increased purifying selection. (**a**) The relationship between MAF and importance scores for microglia, neurons, oligodendrocytes and astrocytes. The *y*-axis shows the average importance scores. Pearson *r*${}^{2}$ values measuring the linear relationships are annotated in plots. Variants and their MAFs were obtained from control samples in gnomAD v2.1.1. Positions not observed in gnomAD were excluded from the analysis. (**b**) The percentage of SNPs in each category that are singletons (Grey = all sites, gold = positions with top 5% importance scores and red = positions with top 0.5% importance scores). In both plots, error bars show ±1 standard error

We hypothesized that variants with high impacts on brain regulatory activity would tend to be deleterious and thus kept at low frequencies in the population. To test this hypothesis, we obtained minor allele frequencies (MAFs) of all SNPs scored by Grad-CAM from the gnomAD (Karczewski *et al.*, 2020) database. We found that in all cell types, rare variants (0 < MAF $<10^{-4}$) have higher average importance scores (Fig. 3a) and that importance scores generally decrease as a function of MAF. Moreover, we stratified these SNPs into three groups based on the ATAC-seq signals (low, medium and high) of their local context regions and found that the correlations between MAF and our importance scores remain strong. For astrocytes, oligodendrocytes and neurons, the Pearson *r*${}^{2}$ scores remain approximately the same regardless of ATAC-seq signals; for microglia, the Pearson *r*${}^{2}$ is strongest in high ATAC-seq signals (Supplementary Fig. S5). We also found that on average importance scores are higher in regions with the highest ATAC-seq signals.

We additionally examined the percentage of variants in different Grad-CAM score bins that are singletons, meaning the variant has only been observed in a single individual. This ‘percent singletons’ has been previously used as a proxy for the deleteriousness of different variant categories (Lek *et al.*, 2016). Variants with top-scoring importance scores (top 5% of Grad-CAM scores) show significantly higher singleton percentages (Z-test for proportions two-sided *P* = $6.04*10^{-27}$). This trend is further pronounced when restricted to the top 0.5% of high-scoring variants (Fig. 3b; *P* = $3.48*10^{-7}$). Overall, variants with high impacts on neuron enhancer activity show the strongest signals of purifying selection, and microglia showed the lowest. Taken together, these results suggest that variants with high impacts on brain enhancer activity are deleterious and are likely targeted by purifying selection.

### 2.4 Linking high-scoring variants with brain traits and disorders

Previous studies have demonstrated an enrichment between cell-type-specific brain enhancers and various neurological and psychiatric disorders (Nott *et al.*, 2019). To investigate whether variants predicted to disrupt enhancer activity might contribute to brain-related complex traits, we analyzed GWAS summary statistics for eight traits and disorders, including AD, schizophrenia, major depressive disorder, bipolar disorder, autism spectrum disorder, intelligence, risky behaviors and insomnia (Supplementary Table S3). For each trait or disorder, we focused on the cell types for which previous analyses (Nott *et al.*, 2019) identified enrichment of trait-associated variants in enhancer regions (Supplementary Table S4). We first applied fine-mapping to restrict our analysis to variants with statistical evidence of causality. To this end, we applied FINEMAP (Benner *et al.*, 2016) separately at each previously identified genome-wide significant locus to identify candidate causal variants for each trait [defined as inclusion in 95% credible sets for each locus and posterior inclusion probability (PIP) >1%]. Importantly, this analysis only considers polymorphic sites (SNPs) as candidate causal variants, and excludes sites with high Grad-CAM scores that are not actually variable in the population. Notably, we did not apply annotation-based fine-mapping tools (Kichaev *et al.*, 2014; Pickrell, 2014) since our importance score annotations only account for a small subset of variants and thus are not amenable to these methods which attempt to learn the importance of each annotation directly from the data. For each trait, on average 30 (1.12%) of all candidate causal variants identified overlapped an H3K27ac-enriched region in the cell types considered for that trait. Of those, we defined high-impact variants as those with importance scores in the top 20th percentile in each region. In total, our pipeline identified 63 distinct fine-mapped variants predicted to influence enhancer activities (Supplementary Tables S4 and S5). In Supplementary Table S5, in addition to our importance scores, we also computed and listed *in silico* mutagenesis scores for each variant (Section 4).

**Fig. 4.**
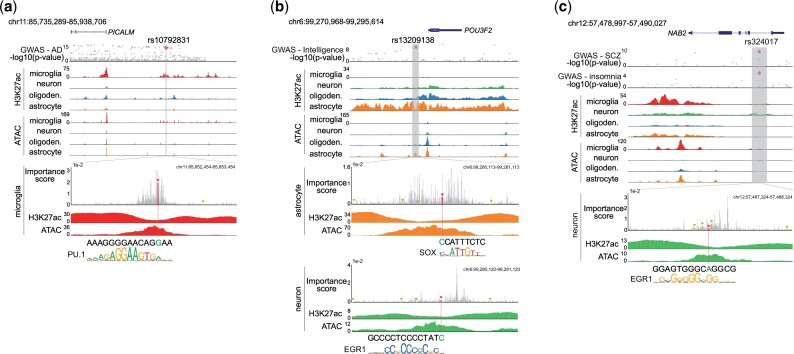
Examples of candidate causal SNPs predicted to impact cell-type-specific brain enhancer activity. In each (**a**–**c**), panels from the top show (i) gene annotations, (ii) GWAS summary statistics (*y*-axis: -log10 *P*-values; *x*-axis: genomic coordinates), (iii) H3K27ac and ATAC-seq ChIP-seq signals for the entire region, (iv) importance scores for sequences surrounding the variant of interest and (v) matched TF motifs

For AD, we identified seven such SNPs. For example, we identified a single SNP (rs10792831) at a strong GWAS signal for AD with FINEMAP PIP = 100% and an importance score in microglia higher than 99.4% of nearby positions (Fig. 4a). This SNP is located ∼74 kb upstream of *PICALM* (Fishilevich *et al.*, 2017), a gene known to affect AD risk primarily by modulating the production, transportation, and clearance of *β*-amyloid (A*β*) peptide (Xu *et al.*, 2015). The SNP disrupts a PU.1 motif in a microglia-specific super-enhancer region (Fig. 4a) previously shown to frequently interact with *PICALM* through long-range chromosomal interactions (Schmitt *et al.*, 2016). Further, this SNP is associated with an allelic imbalance in microglia ATAC-seq (two-sided *P* = $7.28*10^{-4}$) and with *PICALM* expression and splicing (Schubert *et al.*, 2015) in the hippocampus and dorsolateral prefrontal cortex tissues (Section 4; Supplementary Table S6). Additional examples of high-impact SNPs identified in AD include rs10933431 overlapping an intron of *INPP5D* (PIP = 46.4%, ATAC allelic imbalance *P*-value = $5.81*10^{-6}$) and rs7920721 upstream of *USP6NL* (PIP = 14.2%, ATAC allelic imbalance *P*-value = $4.92*10^{-5}$; Supplementary Fig. S6). In both of these cases, the identified SNP overlaps a microglia-specific enhancer region, consistent with the known role of this cell type in AD. Additional cell-type-specific examples are shown for more traits and cell types in Figure 4 and Supplementary Figure S6.

Some of the variants we identified are associated with multiple cell types or traits. For example, SNP rs13209138 (PIP = 34.0% for intelligence) is predicted to impact enhancers with strong H3K27ac signals in both neurons and astrocytes. This SNP overlaps with both the TF motifs of EGR1 and SOX, which are respectively enriched in neurons and astrocytes according to our TF-MoDISco results. This SNP is located upstream of the gene *POU3F2*, a gene known to be important for neuro-differentiation (Pang *et al.*, 2011) (Fig. 4b). In another example, SNP rs324017 is implicated separately in schizophrenia (PIP = 36.8%) and insomnia (PIP = 11.9%; Fig. 4c). This SNP disrupts a binding site for EGR1, a transcription factor involved in response to stress and synaptic plasticity during REM sleep (Duclot and Kabbaj, 2017; Lane *et al.*, 2019).

## 3 Discussion

In this study, we presented a machine learning framework to quantify the contribution of individual genetic variants to brain enhancer activities. Our models can predict cell-type-specific H3K27ac-enriched enhancers directly from sequences with high accuracy (mean auROC = 0.966, mean auPRC = 0.967), and *post hoc* model interpretation using Grad-CAM identified key sequence features driving these predictions. Nucleotide-level importance scores computed by our framework for H3K27ac-enriched enhancers are highly concordant with observed allelic bias in ATAC-seq data. We used these nucleotide-level importance scores to identify 63 candidate variants that may be causally driving published GWAS signals for eight brain-associated traits.

Variants predicted most strongly by our framework to impact brain enhancer activity show signals of increased purifying selection compared to low-scoring variants based on allele frequencies in the general population. This signal is strongest for variants predicted to impact neuron enhancers, and weaker in other cell types, with microglia showing the lowest signal. We hypothesize that this is due to the different roles of these cell types at different stages of life. Disorders for which neurons are implicated as the key cell type, such as autism or other psychiatric disorders, tend to affect individuals early in life or during child-bearing years, and therefore will be more strongly selected against. On the other hand, microglia have been primarily implicated in neurodegenerative diseases (Deczkowska *et al.*, 2018) which tend to occur later in life, and thus may not be subject to negative selection.

While our pipeline identified multiple strong candidate variants, we ultimately found that only a minority of GWAS signals for these traits could be explained by our predictions. Of fine-mapped variants, <1% were sufficiently close to an H3K27ac peak to be scored. The 63 variants in our candidate set were found in 55 distinct GWAS loci, representing 6% (55 out of 910 loci for the eight brain-related traits) of all GWAS signals considered. We suggest several hypotheses for this lack of overlap. First, our analysis is dependent on fine-mapping results. Especially in regions with high LD, fine-mapping may fail to accurately pinpoint the true causal variant. Some existing methods, such as PAINTOR (Kichaev *et al.*, 2014), can incorporate functional annotations to prioritize functionally relevant variants. However, it is challenging to use our importance scores in this way as only a small subset of variants are being studied, leaving 99.3% of variants unannotated. Second, our pipeline considers only the effects of SNPs but is not currently able to score the impact of more complex structural or repetitive variants. Third, our analysis of purifying selection suggests that variants with high impacts on brain enhancer activity are under increased selective pressure, and thus are kept at low frequencies in the population. Thus, they may not be detected by current GWAS datasets, which are mostly powered to detect common variant effects. Fourth, the enhancer data we use are from pediatric patients and do not include individuals from minority populations, and therefore some disease-specific enhancers are likely not included in this dataset. Finally, while it is known that heritability for these traits is enriched in brain-specific regulatory regions, it is possible that some GWAS signals are driven by alternative mechanisms unrelated to enhancer activity or from cell types not interrogated in our study.

Altogether, our study provides a valuable deep learning pipeline that accurately models brain cell-type-specific enhancer activity directly from sequences, and can provide plausible interpretations of strong GWAS signals in multiple traits including AD and schizophrenia. Our framework is easily generalizable, and could also be applied to study the impact of genetic variation on other molecular phenotypes or to characterize the impact of both germline and somatic variants including those arising in cancer. Future efforts will be required to incorporate additional disease-relevant datasets from more diverse groups, and to disentangle the many GWAS signals from these traits that remain unexplained by current variant annotation and fine-mapping frameworks.

## 4 Methods

### 4.1 Brain cell-type-specific ChIP-seq and ATAC-seq datasets

We previously generated ATAC-seq and H3K27ac ChIP-seq data (Nott *et al.*, 2019) for four brain cell types: microglia, neurons, astrocytes and oligodendrocytes. These data were generated from nuclei isolated from the cortical brain tissue of six male individuals with ages ranging from 4 to 18 years. These data can be accessed on dbGap under accession phs001373.v2.p2. We mapped these data to the hg19 genome using Bowtie2 (Langmead and Salzberg, 2012) v2.3.5 with default parameters. Since every cell type has at least three biological replicates of different individuals, we first called unfiltered ATAC-seq peaks for each replicate using the findPeaks script of HOMER (Heinz *et al.*, 2010) v4.11.1 with parameters ‘-style factor -L 0 -C 0 -fdr 0.9 -size 200’. We then used IDR (Li *et al.*, 2011) v2.0.3 with a threshold of 0.05 to identify reproducible open chromatin regions. Since IDR works with only two replicates at a time, we applied it to each pair of replicates of the same cell type and merged the reproducible peaks of each pair of replicates using the mergePeaks script of HOMER with the parameter ‘-d 200’ to reach a final set of reproducible ATAC-seq peaks for every cell type. We computed the normalized number of H3K27ac ChIP-seq tags in an expanded region of 1000 bp centered at each of these peaks and added genomic annotations to these regions using the annotatePeaks.pl script of HOMER with parameters ‘-norm 1e7 -size -500,500’. Normalized tag counts were averaged across replicates of the same cell type. We finally selected a high-confidence set of enhancers for each brain cell type by restricting ATAC-seq peaks to be within intronic or intergenic regions based on HOMER annotations and restricted to peaks with more than 20 averaged, normalized tags of H3K27ac. Our processing step resulted in 21 415 enhancers for microglia, 12 074 enhancers for neurons, 15 774 enhancers for astrocytes and 16 034 enhancers for oligodendrocytes (Supplementary Table S1).

### 4.2 Model training

The sequences of high-confidence enhancers identified above were used as positive sequences in our model training. Negative sequences were chosen to have matched repeat and GC content and were generated using the ‘genNullSeqs’ function of gkmSVM (Ghandi *et al.*, 2016) v0.81.

To avoid the bias of model training toward open chromatin regions and to incorporate wider contexts, we created 10 copies for each positive and negative sequence with equally distanced window shifts with a gap of 100 bp. All copies included the core H3K37ac regions but with different amounts of context areas included upstream and downstream.

Both positive and negative sequences were one-hot encoded into 1000 by 4 matrices based on the sequence present in the hg19 reference genome. Nucleotides marked as ‘N’ were converted to vectors with entries of 0.25 for each of the four nucleotides.

### 4.3 ResNet model architecture

Our ResNet architecture consisted of five standalone convolutional layers, eight residual blocks and two fully connected layers. The standalone convolutional layers have kernels with sizes ranging from 1 to 5 and a number of channels ranging from 64 to 256. The standalone convolutional layers have small kernels: the kernel size of the first two layers is 5, with 128 channels; the kernel size of the next two layers is 3, with 256 channels. The fifth layer is used for dimensionality reduction and has a kernel size of 1 with 64 channels. These convolutional layers are used for extracting basic sequence features such as motifs and motif combinations. The eight residual blocks were constructed the same as in ChromDragoNN (Nair *et al.*, 2019), with a standalone convolutional layer after every two blocks. Batch normalization layers were used after all convolutional layers. The final two layers were fully connected layers with 1000 neurons each. This model takes one-hot encoded 1000 bp sequences as input and outputs a number ranging between 0 and 1 to indicate whether the sequence is predicted to contain an H3K27ac signal.

Previous studies (El Jurdi *et al.*, 2021; Liu *et al.*, 2018; Zhu and Kim, 2021) have shown that adding hard-coded channels for the data coordinates into the convolutional layers can improve their translation invariance property and boost model performance in pattern localization and object detection tasks. In our ResNet model, we converted the first convolutional layer into a CoordConv layer. The coordinates were defined as the distance from the center of H3K27ac regions, with upstream nucleotides labeled as negative and downstream labeled as positive. These coordinates were then re-scaled to range from −1 to 1.

### 4.4 Model interpretation

We implemented the Grad-CAM method (Selvaraju *et al.*, 2017) to interpret our ResNet model by computing an individual score for each nucleotide of the input sequence which indicates its importance in determining the model’s prediction. In our implementation of Grad-CAM, we chose the second to the last standalone convolutional layer prior to the ResNet blocks as the layer of interest. The receptive field of neurons is 13 in the feature maps of this layer, with enough length to cover the cores of most of the common transcription factor motifs. Following the weighting method proposed in the Grad-CAM method, we calculated the weight of each feature map in this layer and used these weights to compute a weighted combination of feature map activations. This gave us a coarse importance map for the input sequence. To acquire a finer resolution at the base-pair level, we mapped this coarse importance map onto the input sequence and multiplied it with input gradients elementwise. We define the importance scores used in downstream analyses as the scores in the resulting finer resolution map. We also computed in-silico mutagenesis scores for the variants of interest by calculating the difference of our ResNet network outputs with the reference sequences and mutated sequences as input.

### 4.5 Benchmarking data augmentation

To understand the influence of augmenting training data through window-shifting, we trained two sets of models, respectively using data with and without window-shifting. All other parameters and training settings were the same. We did not use CoordConv in either of these experiments to avoid introducing implicit position information. After these two sets of models were fully trained, we used them to interpret a test dataset processed by window-shifting. The core H3K27ac regions in this test dataset appeared in each part of the 1000 bp sequence window at the same frequency. We expected the aggregated importance scores to be distributed evenly as is shown in Supplementary Figure S7a. But the aggregated importance scores predicted by the set of models trained without window-shifting were slightly concentrated near the center of 1000 bp sequence windows (Supplementary Fig. S7b). These suggest that the window-shifting process played an important role in making the translation variance property more robust in deep learning models.

### 4.6 Identifying sequence features predictive of H3K27ac-enriched enhancers

To identify important sequence features, we segmented positive H3K27ac sequences from each cell type into 6-bp sequences (6-mers) using a sliding window. We computed the average importance score of each 6-mer and ranked all 6-mers based on this score. We defined top-scoring sequences as those with the top 1% of scores. Similar to in AgentBind (Zheng *et al.*, 2021), we then performed a Fisher’s Exact Test for each 6-mer to test whether it is enriched in the top-scoring subsequences for that cell type. Tests were performed using the fisher_exact method from the Python scipy.stats library (https://docs.scipy.org/doc/scipy/reference/stats.html). The significantly enriched 6-mers were aligned with known motifs from the JASPAR database (Castro-Mondragon *et al.*, 2022) using Tomtom (Gupta *et al.*, 2007) v5.1.1 to infer the most likely motifs associated with every 6-mer.

We additionally used TF-MoDISco (Shrikumar *et al.*, 2018) v0.5.16.0 to cluster and aggregate the importance scores and recover motifs occurring in the H3K27Ac regions. The core 500 bp of each H3K27ac region and its importance scores were used as input. We use its built-in LaplaceNullDist function to generate null distributions with a sampling size of 10 000. To enable TF-MoDISco to find longer motifs, we set the trim_to_window_size as 500 in its seqlets_to_patterns_factory.

### 4.7 Analysis of variant allele frequencies

We obtained SNP allele frequencies computed across 5192 control samples from gnomAD (Karczewski *et al.*, 2020) v2.1.1. For each cell type, we collected the MAFs for the gnomAD SNPs that were also scored in our H3K27ac dataset. We defined singletons as SNPs whose total allele counts in gnomAD were at least 1000 and for which the reference or alternate allele was observed only once. The singleton ratio of a set of SNPs is defined as the percentage of gnomAD SNPs in this set that are singletons.

### 4.8 Allelic imbalance analysis

We combined the original ATAC-seq data of four different individuals (Nott *et al.*, 2019) with twelve additional ATAC-seq data of ex vivo microglia obtained from previous literature (Gosselin *et al.*, 2017). We first masked the hg19 genome with ‘N’ at positions tested by AD GWAS (Jansen *et al.*, 2019a) and re-mapped all the sixteen datasets to this masked genome using Bowtie2 with parameter ‘–np 0’ meaning no penalty for ‘N’ (Langmead and Salzberg, 2012). Then we counted the number of reads with different alleles at each position using the mpileup tool from samtools (Danecek *et al.*, 2021) v0.1.15 followed by the mpileup2snp function of VarScan (Koboldt *et al.*, 2012) v2.4.3. Read counts for the reference and variant allele at each masked position were compared by a binomial test to identify significant allelic imbalance.

In Figure 2b and Supplementary Figure S4, we focused on the SNPs with allelic imbalance data available and binned them based on their allelic imbalance -log10 *P*-values. We used 9 bins evenly distributed between 0 and 20 with a gap of 2.5. The value of each bin defines its lower bound. We also used the same method to bin the allelic imbalance ratio (-log10) in Supplementary Figure S3 and grouped the SNPs into 9 bins evenly distributed between 0.4 and 2.0 with a gap of 0.2. The allelic imbalance ratio is computed as min(total_ref_reads, total_var_reads)/total_reads at each SNP.

### 4.9 Fine-mapping published GWAS signals

We used FINEMAP (Benner *et al.*, 2016) v1.4 to fine-map variants in each genome-wide significant locus identified in the studies listed in Supplementary Table S3. LD for each pair of input variants was computed using the script ‘CalcLD_1KG_VCF.py’ from PAINTOR v3.05 based on available genotypes from the 1000 Genomes Project phase 3 (ftp://ftp.1000genomes.ebi.ac.uk/vol1/ftp/release/20130502/).

Sources of GWAS summary statistics are listed in Supplementary Table S3 (Grove *et al.*, 2019; Jansen *et al.*, 2019a,b; Karlsson Linnér *et al.*, 2019; Mullins *et al.*, 2021; Savage *et al.*, 2018; Skene *et al.*, 2018; Wray *et al.*, 2018). For studies only providing lead SNPs instead of a range, we defined a GWAS locus as a window of 250 000 bp with a lead SNP in the center. FINEMAP was run with default parameters allowing up to 5 causal SNPs per locus.

## Supplementary Material

vbad002_Supplementary_Data

## Data Availability

The data underlying this article, nucleotide-level importance scores, as well as code for running this deep learning pipeline, are available at https://github.com/Pandaman-Ryan/AgentBind-brain.
