# Supplementary material for: Deep learning predicts the impact of regulatory variants on cell-type-specific enhancers in the brain
Source: Bioinform Adv. 2023 Jan 12;3(1):vbad002. doi: 10.1093/bioadv/vbad002 (PMC9887460; doi:10.1093/bioadv/vbad002)
Supplement: vbad002_Supplementary_Data [file vbad002_supplementary_data.zip › vbad002_Supplementary_Data/BrainAgentBind-MainText-supp.pdf]

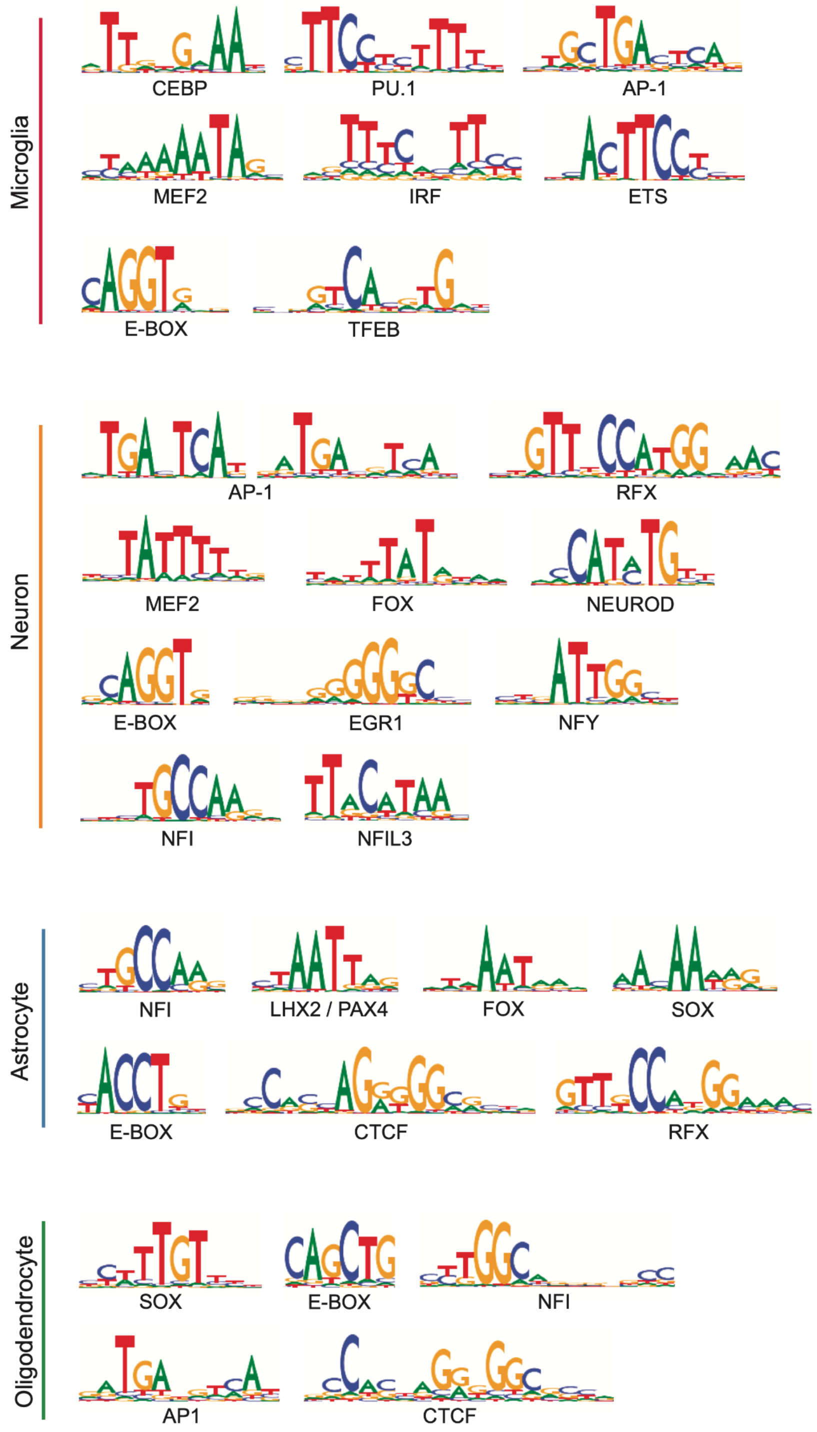

Supplementary Figure 1: Enriched motifs identified by TF-MoDISco for four brain cell types. Motifs were inferred by TF-MoDISco using importance score profiles as input. For each cell type, we list all the motifs identified by TF-Modisco that occur in >1% of the H3K27ac regions. Tomtom[21] was used to associate each motif with known motifs in the JASPAR dataset. We omit redundant motifs and motifs corresponding to low complexity repeat regions.

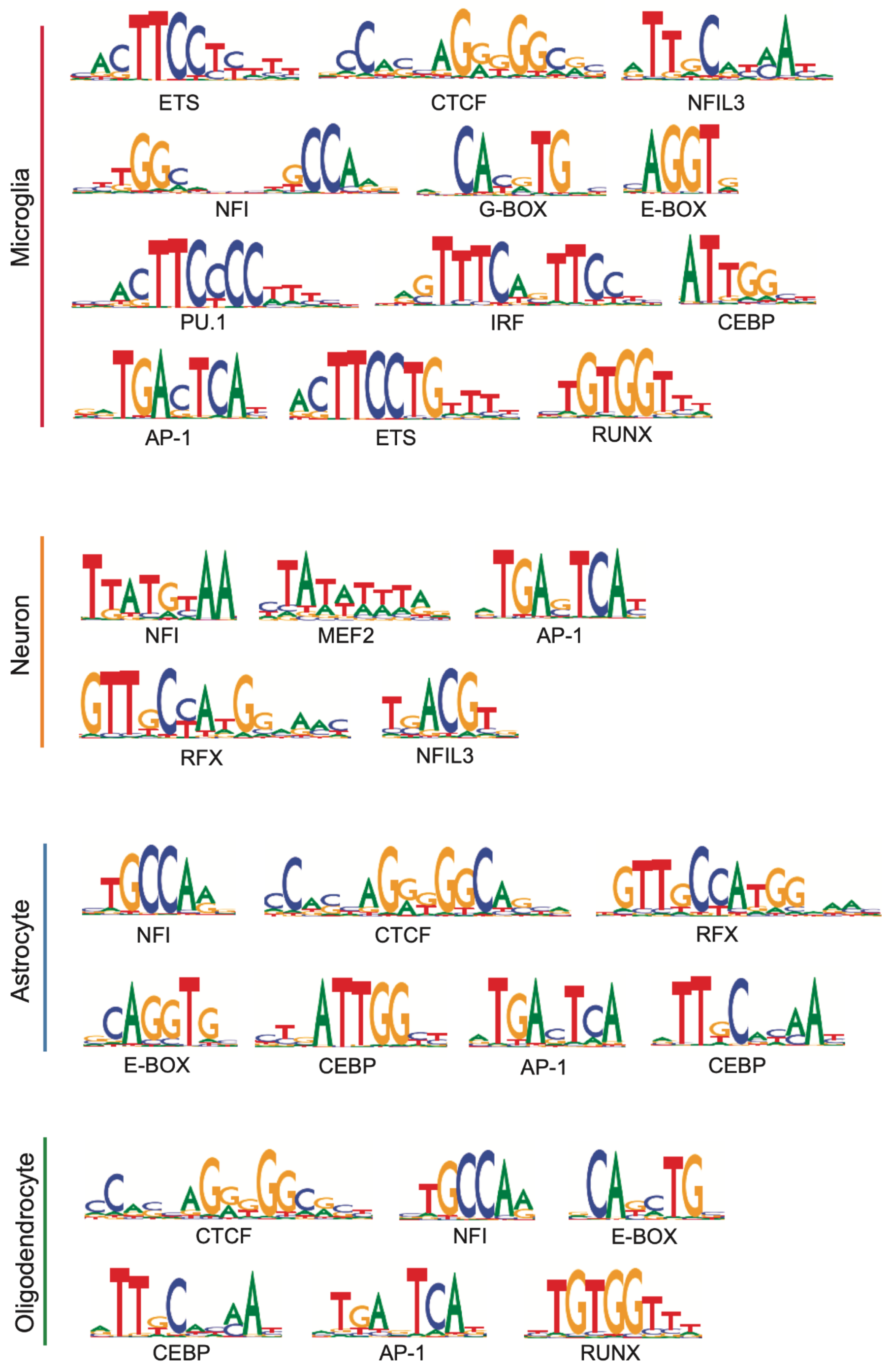

Supplementary Figure 2: Enriched motifs identified by TF-MoDISco for four brain cell types with both positive and negative data in open chromatin regions. As in Extended Data Fig. 1, these motifs were inferred by TF-MoDISco using importance score profiles as input. For each cell type, we list all the motifs identified by TF-Modisco that occur in >1% of the H3K27ac regions. Tomtom[21] was used to associate each motif with known motifs in the JASPAR dataset. We omit redundant motifs and motifs corresponding to low complexity repeat regions.

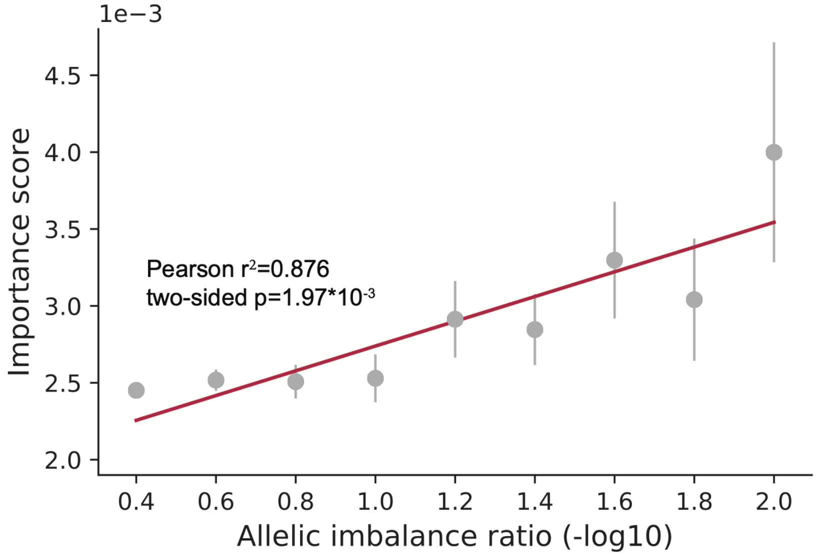

Supplementary Figure 3: Comparison of the allelic imbalance ratio vs. importance scores inferred by our deep learning models. The allelic imbalance ratio for each SNP was computed based on microglia ATAC-seq. We grouped SNPs by the degree of allelic imbalance ratio (**Methods**) and computed the average importance score for each group. Error bars give +/- 1 standard error.

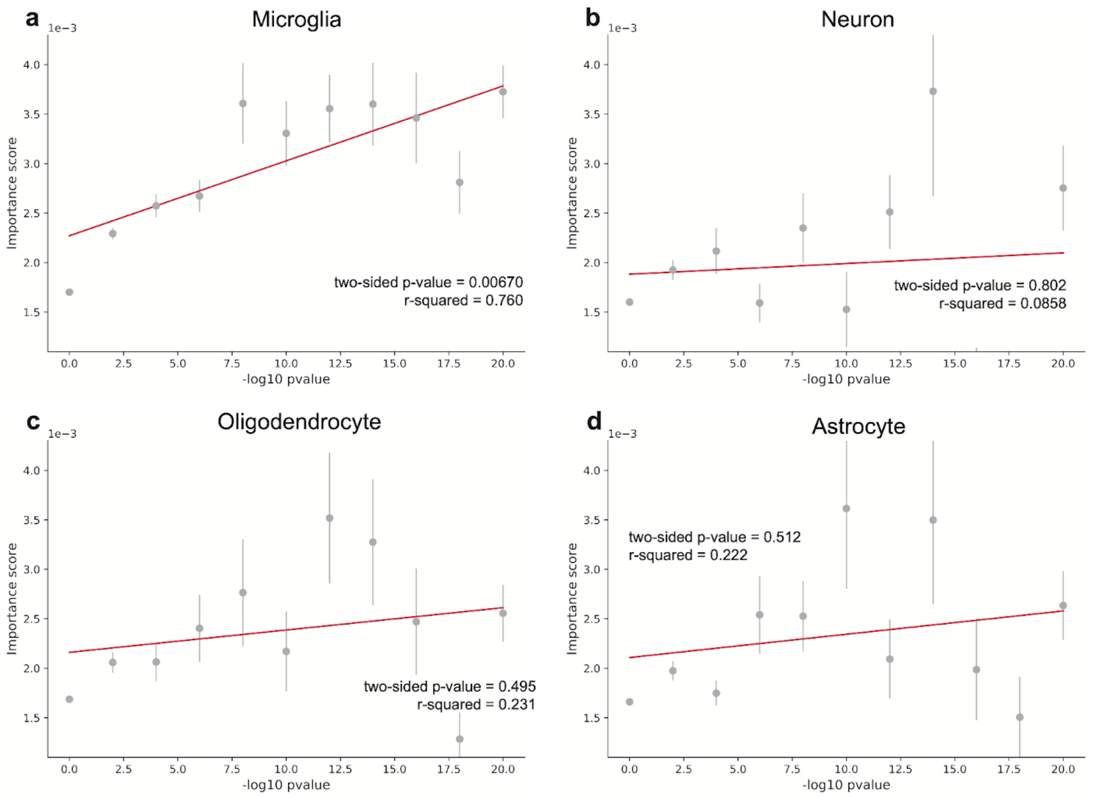

Supplementary Figure 4: Comparison of allelic imbalance p-values vs. importance scores inferred by our deep learning models. The allelic imbalance score for each SNP was computed based on microglia ATAC-seq. We grouped SNPs by -log10 allelic imbalance p-values (**Methods**) and computed the average importance score for each group of SNPs. Panels show allelic imbalance compared to scores computed from models for (a) microglia, (b) neurons, (c) oligodendrocytes, and (d) astrocytes. Error bars give +/- 1 standard error. As expected, scores learned from the microglia model are most concordant with allelic imbalance data from microglia.

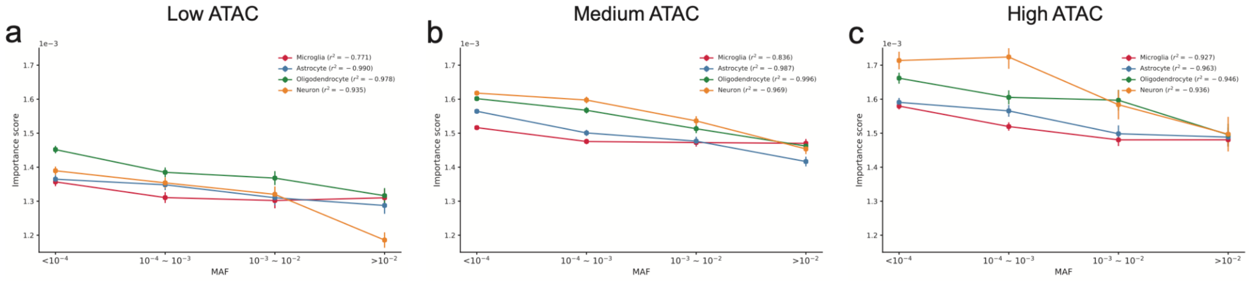

Supplementary Figure 5: Variants predicted to have high impacts on brain enhancer activity are under increased purifying selection. (a)-(c) The relationship between MAF and importance scores for microglia, neurons, oligodendrocytes, and astrocytes with SNPs overlapping with (a) low ATAC regions (signal intensity < 20); (b) medium ATAC regions (signal intensity between 20 to 80); and (c) high ATAC regions (signal intensity > 80). The y-axis shows the average importance scores. Pearson r^2 values measuring the linear relationships are annotated in the plots. Variants and their MAFs were obtained from control samples in gnomAD v2.1.1. Positions not observed in gnomAD were excluded from the analysis.

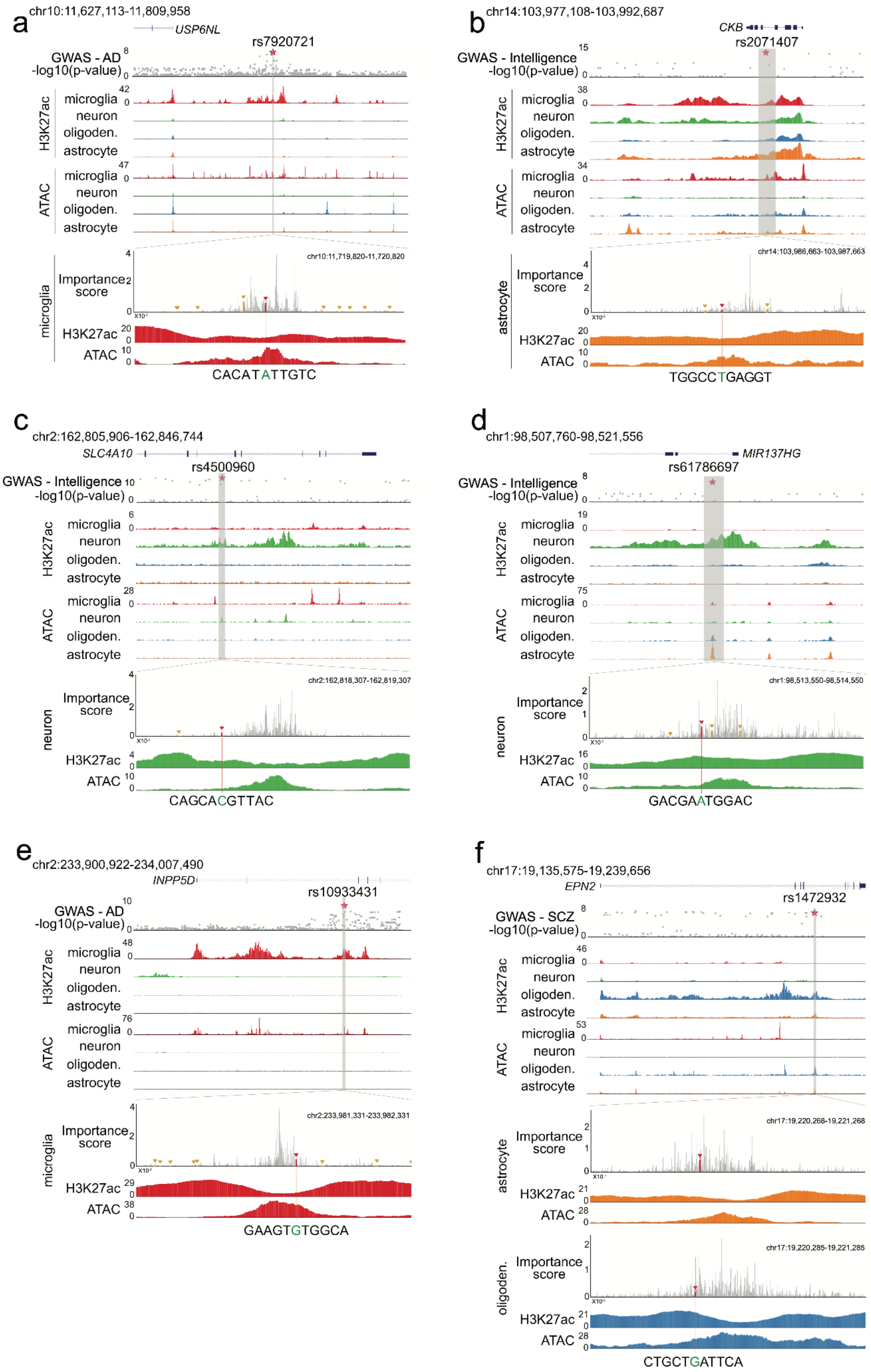

a
chr10:11,627,113-11,809,958
USP6NL
rs7920721
GWAS - AD
-log10(p-value)
H3K27ac
ATAC
microglia
neuron
oligoden.
astrocyte
chr10:11,719,820-11,720,820
Importance score
CACATATTGTC
b
chr14:103,977,108-103,992,687
CKB
rs2071407
GWAS - Intelligence
-log10(p-value)
chr14:103,986,663-103,987,663
TGGCCTGAGGT
c
chr2:162,805,906-162,846,744
SLC4A10
rs4500960
chr2:162,818,307-162,819,307
CAGCACGTTAC
d
chr1:98,507,760-98,521,556
MIR137HG
rs61786697
chr1:98,513,550-98,514,550
GACGAATGGAC
e
chr2:233,900,922-234,007,490
INPP5D
rs10933431
chr2:233,981,331-233,982,331
GAAGTGTGGCA
f
chr17:19,135,575-19,239,656
EPN2
rs1472932
GWAS - SCZ
chr17:19,220,268-19,221,268
chr17:19,220,285-19,221,285
CTGCTGATTCA

Supplementary Figure 6: Examples of candidate putative causal SNPs predicted to impact cell-type-specific brain enhancer activity. In **(a)-(c)**, panels from the top show (1) gene annotations, (2) GWAS summary statistics (y-axis: -log10 p-values; x-axis: genomic coordinates), (3) H3K27ac and ATAC-seq ChIP-seq signals for the entire region, and (4) Importance scores for sequences surrounding the variant of interest.

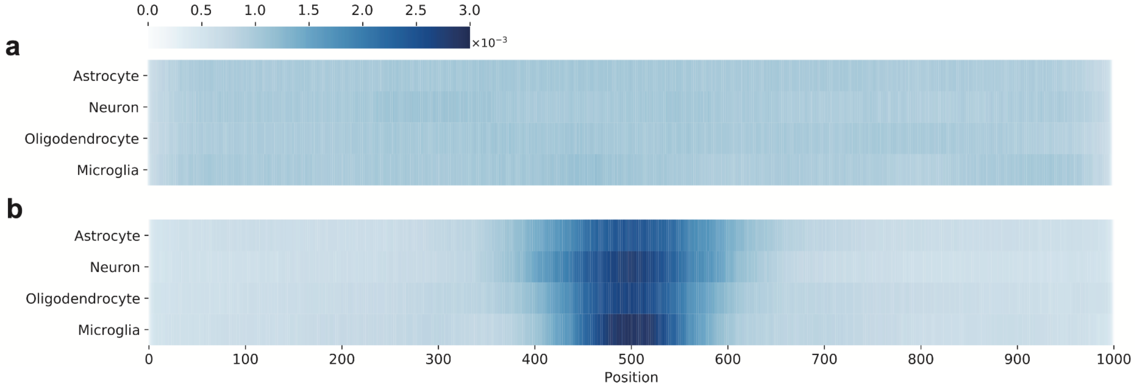

Supplementary Figure 7: Evaluating the robustness of important scores to window-shifting. (a) and (b) show aggregate importance score profiles, computed as the average absolute value of the importance score at each position. In (a), importance scores were predicted using a model trained on augmented training datasets containing shifted versions of the original sequences, whereas in (b), importance scores were predicted using a model trained on the original training datasets without window-shifting.
